# Draft genome sequences of *Corynebacterium mastitidis* strains isolated from ocular surface of CD36-knockout mice (B6.129S1-*Cd36^tm1Mfe^*/J) with keratitis

**DOI:** 10.1128/mra.00562-24

**Published:** 2024-12-12

**Authors:** Anthony Mannion, Zeli Shen, Ellen Buckley-Jordan, Alexis Garcia, Claire Lyons, Yao Lee, Magalie Boucher, Sebastian Carrasco, Michael S. Gilmore, James G. Fox

**Affiliations:** 1 Division of Comparative Medicine, Massachusetts Institute of Technology, Cambridge, Massachusetts, USA; 2 Departments of Ophthalmology, and Microbiology and Immunobiology, Harvard Medical School, Massachusetts Eye and Ear Infirmary, Boston, Massachusetts, USA; University of Maryland School of Medicine, Baltimore, Maryland, USA

**Keywords:** CD36-knockout mice, keratitis, *Corynebacterium mastitidis*, whole genome sequencing, virulence factors, phylogenetics

## Abstract

Three *Corynebacterium mastitidis* strains were cultured from the eyes of CD36-knockout mice (B6.129S1-*Cd36^tm1Mfe^*/J) with and without keratitis housed at a biomedical research institute. Bacteria were sequenced using Illumina MiSeq technology for subsequent phylogenetic characterization and identification of virulence factor genes conferring pathogenic potential.

## ANNOUNCEMENT

*Corynebacterium* species are Gram-positive, aerobic bacteria that colonize the skin and other external surfaces of mammals, including humans and mice. Several *Corynebacterium* species have been implicated with infectious diseases in susceptible hosts, such as dermatitis in immunocompromised mice. Recently, *Corynebacterium mastitidis* (*Cm*) has been cultured from the conjunctiva of research mice with microphthalmia, blepharitis, and keratitis (1–3). Herein, we describe the isolation of 16 *Cm* strains from the eyes of 10 CD36-knockout mice, a majority with keratitis, housed in an AAALAC-accredited vivarium. CD36 encodes a Class B scavenger receptor constitutively expressed on the corneal epithelium that regulates multiple homeostatic and pathogenic functions (4, 5). Whole genome sequencing was performed on three representative *Cm* isolates (two from eyes with keratitis and one from normal eye) for phylogenetic characterization and identification of virulence factor genes that may confer pathogenic potential.

Eye swabs aseptically collected from necropsied mice were cultured at 37°C under aerobic conditions on MacConkey blood agar media for 14 days, according to procedures approved by MIT’s Committee on Animal Care (i.e., IUCAC). Genomic DNA from individual colonies was isolated using the High Pure PCR Product Purification Kit and was identified as *Cm* by PCR amplification and sequencing of the 16S rRNA gene, using methods described previously (6). Genomic DNA was prepared for Illumina MiSeq sequencing (2 × 150 bp) by the BioMicroCenter at MIT using the Nextera Flex library prep kit. Default parameters were used for all bioinformatics tools. Raw sequence reads were decontaminated of adapters and quality trimmed (Trim Galore version: 0.6.5dev and Cutadapt version: 4.2) followed by *de novo* contig assembly (Unicycler version: 0.4.8) and gene annotation (RASTtk version: 2020-04-06) via the BV-BRC Comprehensive Genome Analysis tool (7). Genome summary statistics are described in Table 1.

**TABLE 1:** Summary genome statistics

Isolate accession	Isolation tissue	No. of contigs	N50 (bp)	Coverage (×)	Genome size (bp)	GC content (%)	Predicted no. of: ^*c*^			Total no. of reads		GenBank accession no.	SRA accession no.	16S rRNA accession no.
							Proteins	tRNAs	rRNAs	Before quality control	After quality control			
MIT 2212200033^*a*^	CD36-knockout mouse eye with keratitis	55	64374	149.7	2208133	69.1	2172	51	2	984860	983910	JBBMMI000000000	SRR28480439	PQ110286
MIT 2201300027^*a*^	Normal CD36-knockout mouse eye	142	26546	181.6	2180940	69.0	2245	51	2	1183904	1182366	JBBMMH000000000	SRR28480438	PQ110287
MIT 2301260027^*a*^	CD36-knockout mouse eye with keratitis	41	102791	216.8	2215776	69.2	2170	51	2	1433118	1430420	JBBMMG000000000	SRR28480437	PQ110288
16-1433^*b*^	Mouse skin	65	61179	197.8	2264319	69.0	2291	52	1	n/a^*d*^	n/a	PJAF00000000	n/a	n/a
RC^*b*^	Mouse conjunctiva	42	158766	89.0	2153054	69.1	2130	53	2	n/a	n/a	JAKRKB000000000	n/a	n/a
DSM 44356^*b*^	Milk of sheep with subclinical mastitis	39	243176	n/a	2370005	69.0	2166	48	5	n/a	n/a	AQXB00000000	n/a	n/a
MSK081^*b*^	Nasopharyngeal swab from human	19	317260	106.0	2368744	68.7	2409	51	2	n/a	n/a	JASPIQ000000000	n/a	n/a

^*a*^ Current study.

^*b*^ Accessed from BV-BRC database.

^*c*^ Number of gene sequences predicted using RASTtk via the Comprehensive Genome Analysis tool hosted by BV-BRC.

^*d*^ n/a, not available or applicable.

The phylogenetic similarity of the novel *Cm* strain with other genomes for *Corynebacterium* species available in the BV-BRC database, including four *Cm* genomes, was assessed by average nucleotide identity (ANI) calculated using pyani (version: 0.2.10) (8). The novel *Cm* strains were 99.99% identical to each other. The novel *Cm* strains were 99.47% similar to *Cm* strains 16-1433 (1) and RC (2) isolated from the skin and conjunctiva of research mice as well as 96.69%–96.76% similar to *Cm* strain DSM 44356 cultured from the milk of sheep with mastitis and *Cm* strain MSK081 isolated from a human nasopharyngeal swab. All *Cm* genomes had ANI below 95% versus non-*Cm* genomes, confirming their taxonomic classification.

**Fig 1:**
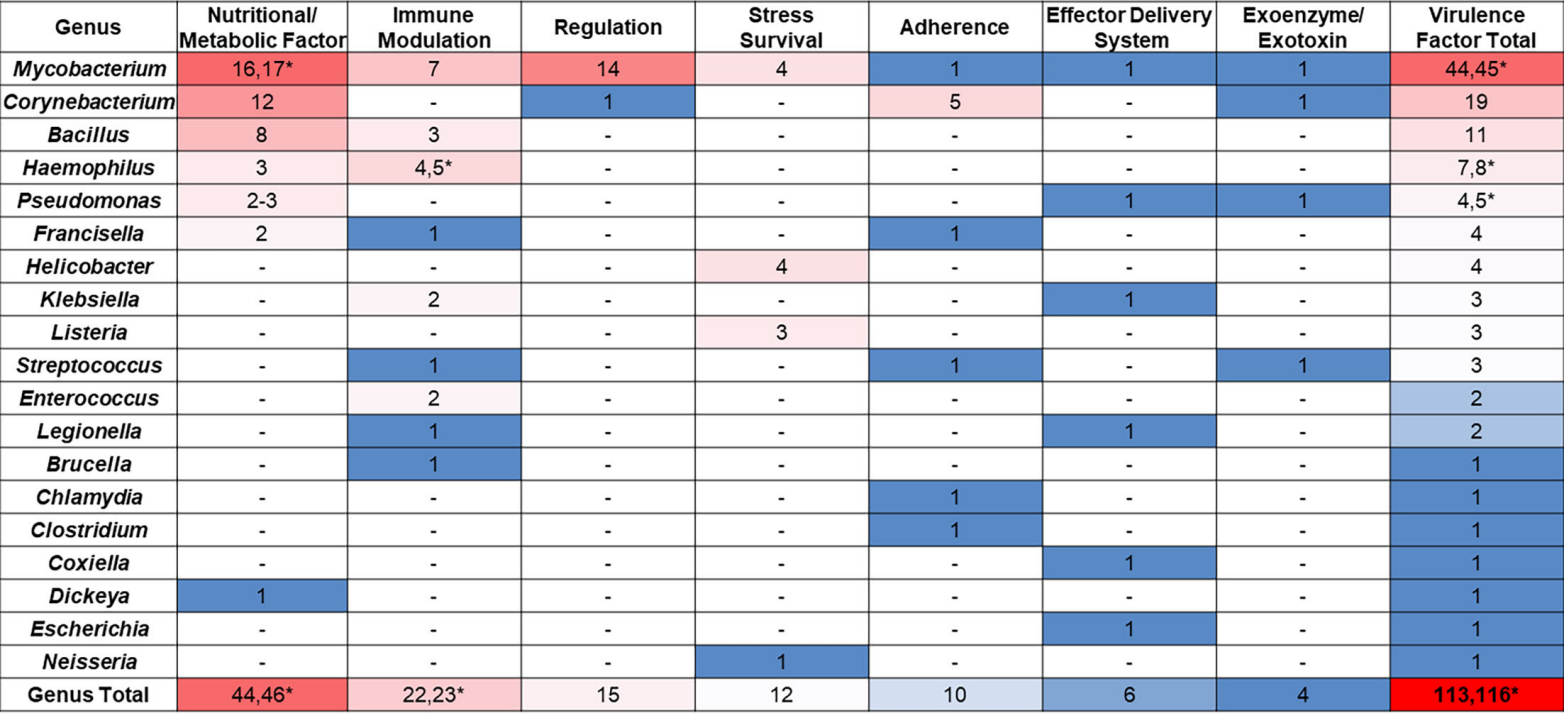
Heatmap showing number of virulence factor gene homologs identified in novel *C. mastitidis* strains MIT 2212200033, MIT 2201300027, and MIT 2301260027 genomes. Columns indicate virulence factor function, and rows indicate genus of the homolog genes. Asterisks (*) indicate extra homolog genes detected in *C. mastitidis* strain MIT 2201300027 genome.

DIAMOND analysis (version: 2.1.9.163; identity, coverage ≥40%; *E*-value ≤0.00001) (9) against the Virulence Factors Database (10) and genes associated with *Corynebacterium* species pathogenicity (11) identified 113-116 virulence factors in the novel *Cm* genomes (Fig. 1). These included iron/heme acquisition, adherence/invasion factors, mycolic acid synthesis for cell wall modification, secreted serine proteases, type IV and six secretion system effectors, and urease genes. Most homologs represented nutritional/metabolic factors from *Mycobacterium* and *Corynebacterium* species (Fig. 1).

In conclusion, our genome analysis suggests that *C. mastitidis* has the pathogenic potential to influence ocular and other diseases in susceptible mouse strains.

## Data Availability

Genomes and sequencing reads as well as 16S rRNA sequences have been deposited in GenBank and SRA database under the accession numbers described in Table 1. Genomes submitted to GenBank were automatically annotated by PGAP.
